# Supplementary material for: Calpain-1 and Calpain-2 in the Brain: New Evidence for a Critical Role of Calpain-2 in Neuronal Death
Source: Cells. 2020 Dec 16;9(12):2698. doi: 10.3390/cells9122698 (PMC7765587; doi:10.3390/cells9122698)
Supplement: Supplementary file 1 [file cells-09-02698-s001.pdf]

Supplementary Materials

Calpain-1 and calpain-2 in the brain: New evidence for a critical role of calpain-2 in neuronal death

Yubin Wang, Yan Liu, Xiaoning Bi and Michel Baudry*

Materials and Methods

Antibodies

Primary antibodies for Western Blot: Spectrin (1:500; MAB1622, EMD Millipore), STEP (1:1000, NB300-202, Novus Biologicals), PTPN13 (1:1000, PA5-72906, Thermo Fisher Scientific), and TDP-43 (1:1000, 10782-2-AP, Proteintech). Primary antibodies for immunohistochemistry: Calpain-2 (1:300; LS-C337641, LSBio), SBDP (1:500, a gift from Dr. Saido, Riken, Japan), and full-length PTEN (1:600, 9556, Cell Signaling).

Controlled cortical impact

CCI model of traumatic brain injury was performed as previously described [1]. Mice (3-month old, 25-30 g) were anesthetized using isoflurane and fixed in a stereotaxic frame with a gas anesthesia mask. A heating pad was placed beneath the body to maintain body temperature around 33-35 ˚C. The head was positioned in the horizontal plane. The top of the skull was exposed, and a 5-mm craniotomy was made using a micro drill lateral to the sagittal suture, and centered between Bregma and Lambda. The skull at the craniotomy site was carefully removed without damaging the dura. The exposed cortex was hit by a pneumatically controlled impactor device (AMS-201, Amscien). The impactor tip diameter was 3 mm, the impact velocity was 3 m/sec, and the depth of cortical deformation was 0.5 mm. After impact, the injured region was sutured using tissue adhesive (3M) and mice were placed in a 37 °C incubator until they recovered from anesthesia. In sham surgery, mice were sutured after craniotomy was performed.

Immunohistochemistry

Mice were anesthetized and perfused intracardially with 0.1 M phosphate buffer (PB, pH 7.4), then with freshly prepared 4% paraformaldehyde in 0.1 M phosphate buffer. Brains were removed and immersed in 4% paraformaldehyde at 4 ºC for one day for post-fixation, then in 15% and 30% sucrose at 4 ºC for one day each for cryoprotection. Coronal frozen sections (20 μm thick) at Bregma 0.74 and -1.58 were collected. Sections were first blocked in 0.1M PBS containing 5% goat or donkey serum and 0.3% Triton X-100 (blocking solution) for 1 h, and then incubated with primary antibody prepared in blocking solution overnight at 4 ˚C. Sections were washed 3 times in PBS and incubated in Alexa Fluor secondary antibody prepared in blocking solution (1:400) for 2 h at room temperature. After three washes, sections were mounted with mounting medium containing DAPI (Vector Laboratories, Burlingame, CA). Sections were visualized under confocal microscopy (ZEISS LSM 880).

Western Blot

Mouse cortical tissue was isolated and placed in ice-cold homogenization buffer containing the following: 320 mM sucrose, 10 mM HEPES, pH 7.4, 2 mM EDTA and protease and phosphatase inhibitor cocktail (Thermo Fisher Scientific). Tissue was homogenized using a glass homogenizer. The homogenate was centrifuged at 1000 x g for 10 min, and the resultant supernatant was collected and centrifuged at 14,000 x g for 20 min. P2 pellets were then resuspended in RIPA buffer (89901, Thermo) with a protease inhibitor cocktail (78446, Thermo). Protein concentrations were measured using the BCA protein assay kit (Thermo). Sample buffer was added, and samples were separated in SDS-PAGE and transferred onto PVDF membranes (IPFL00010, Millipore). After blocking with Odyssey® Blocking Buffer (LiCOR) for 1 h, membranes were incubated with primary antibodies overnight at 4 ˚C and then with secondary antibodies for 2 h at room temperature. Protein bands were visualized with the Odyssey imaging system (LI-COR).

SNTF immunoassay

The sandwich immunoassay for quantifying calpain-cleaved SNTF from mouse plasma was performed as previously described [2]. Briefly, 96 well plastic microplates with an underside electrode (Meso Scale Diagnostics) were coated overnight with the capture antibody, a monoclonal directed at the SH3 domain in the N-terminal portion of the αII-spectrin subunit (D8/B7 @ 1/1000; Abcam). For the antigen capture step, mouse plasma samples diluted to 40% or SNTF standards (25 µls/well) prepared in 0.25% bovine serum albumin in Tris-buffered saline (pH 7.4) were added in triplicate for 2 h at 22°C. The detection antibody was a purified rabbit IgG prepared in our laboratory that is reactive with the calpain-generated neoepitope at the carboxyl-end of the stable calpain-derived α-spectrin ~150 kDa N-terminal fragment (SNTF; 1/5,000). The reporter probe was goat anti-rabbit IgG conjugated to ruthenium (SulfoTag, Meso Scale Diagnostics, Rockville, MD, USA; 1/500). In the presence of read buffer containing tripropylamine and application of current to the plate electrode, a chemiluminescent product is produced in proportion to the bound antigen. Chemiluminescent signals were quantified by a SECTOR Imager 2400 system (Meso Scale Diagnostics). Standard curves were generated using serial dilution of a preparation of α-spectrin partially purified from brain and digested with purified calpain I. One unit of SNTF is defined as the signal derived from the SNTF standard diluted to 1 nl/ml, corresponding to ~500 pg of the spectrin-containing brain extract starting material per ml.

References

1. Wang, Y.; Liu, Y.; Lopez, D.; Lee, M.; Dayal, S.; Hurtado, A.; Bi, X.; Baudry, M. Protection against TBI-induced neuronal death with post-treatment with a selective calpain-2 inhibitor in mice. *Journal of Neurotrauma* **2017**.

2. Siman, R.; Giovannone, N.; Hanten, G.; Wilde, E.A.; McCauley, S.R.; Hunter, J.V.; Li, X.; Levin, H.S.; Smith, D.H. Evidence That the Blood Biomarker SNTF Predicts Brain Imaging Changes and Persistent Cognitive Dysfunction in Mild TBI Patients. *Front Neurol* **2013**, *4*, 190, doi:10.3389/fneur.2013.00190.

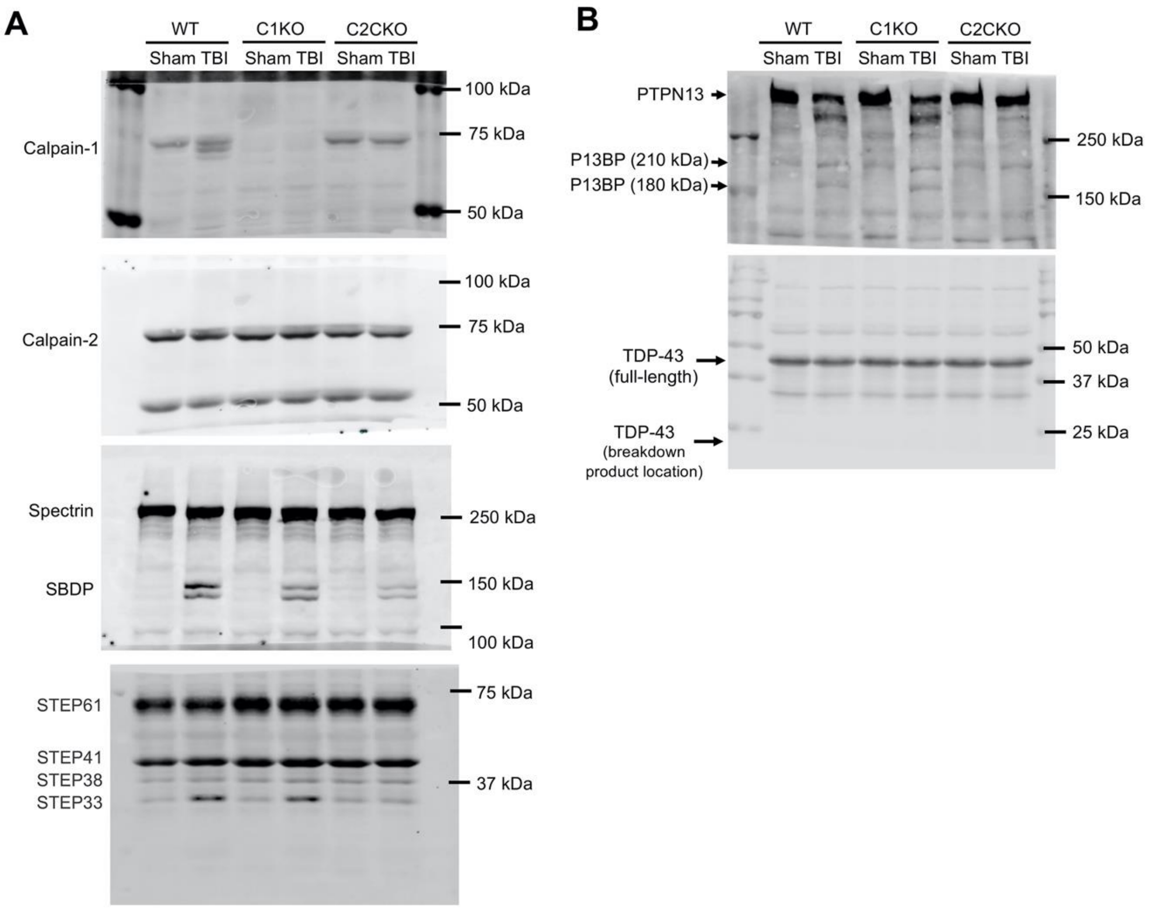

Full-length immunoblots for Figure 2.
